# Enhanced risk of cancer in companion animals as a response to the longevity

**DOI:** 10.1038/s41598-020-75684-4

**Published:** 2020-11-11

**Authors:** Moeko Tanaka, Sachi Yamaguchi, Yoh Iwasa

**Affiliations:** 1 Department of Bioscience, School of Science and Technology, Kwansei Gakuin University, 2-1 Gakuen, Sanda-shi, Hyogo 669-1337 Japan; 2 Division of Mathematical Sciences, Tokyo Woman’s Christian University, 2-6-1 Zempukuji, Suginami-ku, Tokyo 167-8585 Japan

**Keywords:** Applied mathematics, Cancer models, Evolutionary theory

## Abstract

Cancer is caused by the lifetime accumulation of multiple somatic deformations of the genome and epigenome. At a very low rate, mistakes occur during genomic replication (e.g., mutations or modified epigenetic marks). Long-lived species, such as elephants, are suggested to have evolved mechanisms to slow down the cancer progression. Recently, the life span of companion dogs has increased considerably than before, owing to the improvement of their environment, which has led to an increase in the fraction of companion dogs developing cancer. These findings suggest that short-term responses of cancer risk to longevity differ from long-term responses. In this study, to clarify the situation, we used a simple multi-step model for cancer. The rates of events leading to malignant cancer are assumed to be proportional to those of genomic replication error. Perfect removal of replication error requires a large cost, resulting in the evolution of a positive rate of genomic replication error. The analysis of the model revealed: that, when the environment suddenly becomes benign, the relative importance of cancer enhances, although the age-dependent cancer risk remains unchanged. However, in the long run, the genomic error rate evolves to become smaller and mitigates the cancer risk.

## Introduction

Cancer is caused by the lifetime accumulation of multiple somatic deformations of the genome and epigenome^1,2^. During genomic replication, there mistakes occur at a very low rate (e.g., mutations or modified epigenetic marks). In cancer biology, multi-step models for cancer have been adopted to analyze the age-incidence relationship^3,4^. They represent the following idea: a just born wild-type individual is at state 0, and then every time the individual experiences an event, the step number increases by one. When the step number reaches $n$, the individual develops a malignant cancer. Solid tumors normally require the realization of many steps, although leukemia requires a relatively small number of mutations^4–6^. In familial cases, the individual of interest inherits the mutations or other genomic deformation from their parents and has a propensity to develop cancer earlier than in sporadic cases. However, each step of the multi-step model for cancer may not correspond to one mutation. If several errors accumulate very quickly, these steps may appear as a single-step event. Conversely, a single mutation that increases the number of cells and causes malignant cancer may appear to require three or more steps in the age-incidence curve, as shown by mathematical analysis^5^. Hence the contrast between the solid tumors (large $n$) and leukemia (small $n$) could be explained by the step number dependence of the model.

The rate of these errors caused by somatic mutations may slow down if an organism checks the genome replication process more carefully and detects the presence of cancer cells and removes them. This can be noted in some organisms with a very long life. Species that live much longer than other related species in the wild, such as elephants, have evolved mechanisms to slow down cancer progression. For example, p53 is a well-known tumor suppressor that removes cells that are potentially cancerous. Elephants are known to have twenty loci, whereas humans only have a single locus, for the *p53* gene^7,8^. The p53 protein activates the *LIF6* gene, which encodes a leukemia inhibitory factor, LIF, and also functions as a tumor suppressor. LIF is inactive in the normal state; however, when it becomes active in cells that become cancerous, it induces apoptosis of the cells. By removing cells with DNA damage, it prevents the spread of cancer cells in the body. Elephants have active *LIF* genes and acquire the ability to reduce of the risk of cancer^7–9^. Unlike other rodents, capybaras have larger body size and live longer. Capybaras were suggested to have a mechanism to suppress fast-growing cells that are possibly cancerous^10^. These potential examples are plausibly interpreted as the outcomes of natural selection operating on the genomic machinery including replication and error checking mechanisms, according to the life history evolution theory^11^. To apply this argument, we need to analyze the allometric relationships among the longevity, the body size, the total number of cell divisions, and the risk of cancer. Some theoretical analysis of allometric relation among mammals has been developed to evaluate the risk of chromic myeloid leukemia^12,13^.

Recently, the longevity of companion animals has increased, owing to the improvement of their food, shelter, hygiene, and health care. However, this has also increased the proportion of individuals with malignant cancer (neoplasia). For example, at present, the largest mortality factor in aged companion dogs is malignant cancer (neoplasia) in countries including Sweden, the United States, the United Kingdom, and Japan^14–17^. The fraction of cancer as the mortality factor was between 15 and 30%, differing between countries and breeds^16^. This is presumably because of the extension of longevity owing to the improvement of the environment, leading to the mitigation of many mortality factors other than cancer. Hence, a short-term response of the population could be the enhanced importance of cancer, which appears to be opposite to the expected outcome of evolutionary responses of the species to extended longevity.

In this study, to clarify the logic underlying these arguments, we developed a multi-step model for cancer, where the step number progresses when an event occurs in an individual. The rate of events is faster as the genomic error rate increases. Not only the replication steps of the genome, the maintenance of the genome is also accompanied by some change of errors, because of DNA damage, which can be reduced by investing in the machinery of damage repair. In this paper, we consider errors accompanying the genomic replication. We assume that the accurate elimination of errors during genomic replication incurs a large cost. Because natural selection operates on the genomic replication machinery, the population would evolve a small but positive rate of replication error, which would cause some risk of cancer. Natural selection pressure to remove the risk of cancer is stronger for long-lived animals than for short-lived ones. Hence, evolution reduces the cancer risk in long-lived species. Conversely, a species suddenly provided a considerably benign environment than before, with better food availability, lack of physical accidents or predators, better hygiene, and even better medical care, would experience extended longevity, and thus a higher cancer risk than before. This change occurs without the evolutionary responses of the genomic replication machinery. We elucidated the mathematical relationship between long-term (evolutionary) and short-term (non-evolutionary) responses of the population regarding the cancer risk.

## Model and results

### Multi-step model for cancer

**Figure 1:**
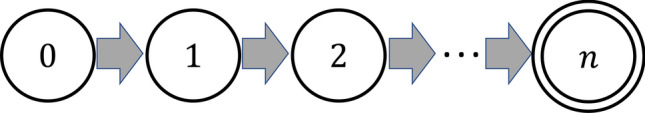
Schematic of the multi-step model (or multistate model) for cancer. Animals were born in step 0. At random times, each individual experiences an "event", and its state number is added by one. An event corresponds to the accumulation of a somatic mutation or a somatic change in epigenetics. When an individual reaches state $n$, it develops malignant cancer.

The simplest phenomenological model for cancer is an $n$-step model that postulates that individuals are born in the 0 step, and they experience multiple events, and, unless they are killed by accidents or other diseases, they eventually develop malignant cancer when they experience $n$ events (Fig. 1)^3^. These events are considered as any progression of the disease and are assumed to occur at random time points. Multi-step models for cancer have been adopted to analyze the age- and sex-dependence of cancer incidence (e.g.,^4^). As the model to describe the process toward cancer, we here adopt the simplest possible one^3^ that describe the state of an individual, instead of those considering the cellular proliferation and cell death, mutations, cell fitness difference, genomic instability, angiogenesis and metastasis. We chose this because the best model to adopt is the simplest one among those that capture the essence of the phenomena.

The mechanism leading to malignant cancer requires diverse events such as the loss of tumor suppressors, mutation in oncogenes, and acquisition of the ability to induce angiogenesis and metastasis. Notably, the number of steps in the multi-step model may not correspond to the number of mutations required for the progression of steps. The step number can be smaller than the real number of mutations because some genomic changes occur considerably faster than others. However, the step number can be larger than the number of mutations because of the time required for the growth of cancer cells may appear as many steps in the incidence-age curve, as illustrated by an example in which a single mutation causing chronic myeloid leukemia may appear as many as three steps^5^.

#### Progression of step number

We consider a simple $n$-step model (*n* > 1). At age zero ($a=0$), all the individuals are in step 0, which is the initial state. As they age, stem cells divide and the genome accumulates mutations and errors in epigenetic marks. These cells divide faster than the others and take over the stem cell population, which makes one step ahead in the model. Let ${c}_{i}$ be the rate of transition from step $i$ to step $i+1$. The step $n$ corresponds to the individual who develops malignant cancer (Fig. 1).

Let ${P}_{i}(a)$ be the probability that an individual will be in state $i$ at age $a$ ($i=\mathrm{0,1},\ldots,n$). The dynamics of the different states are as follows:

1a $$\frac{{dP_{0} }}{da} = - c_{0} P_{0} ,$$

1b $$\frac{{dP_{i} }}{da} = c_{i - 1} P_{i - 1} - c_{i} P_{i} ,\quad \left( {i = 2,{ }3,{ }4 \ldots ,n - {1}} \right)$$

1c $$\frac{{dP_{n} }}{da} = c_{n - 1} P_{n - 1} .$$

The initial condition is ${P}_{0}\left(0\right)=1$ and ${P}_{1}\left(0\right)={P}_{2}\left(0\right)= \cdots {P}_{n}\left(0\right)=0$.

We consider the case in which the rates of transition between states, that is, the rate for an individual to experience the next event, is proportional to the error rate of replication, $x$. In particular, we assume ${c}_{i}={k}_{i}x$ where $i=\mathrm{0,1},2,\ldots,n-1$. By reducing the error rate of genome replication $x$, the species can reduce the speed of progression toward malignant cancer development. We represent unit of error rate per year, and $x$ is heritable.

In the simple case in which the transition rates between states are a common constant ${k}_{0}={k}_{1}=\cdots={k}_{n}=k$, we can obtain the time-dependent solution of Eq. (1) as follows:

2a $$P_{i} \left( a \right) = \frac{{\left( {kx} \right)^{i} a^{i} }}{i!}e^{ - kxa} ,\quad (i = 0,1,2, \ldots ,n - 1)$$

and

2b $$P_{n} \left( a \right) = \frac{1}{{\left( {n - 1} \right)!}}\mathop \int \nolimits_{0}^{kxa} y^{{\left( {n - 1} \right)}} e^{ - y} dy = \frac{1}{{\left( {n - 1} \right)!}}\gamma \left( {n,kxa} \right),$$

where $\gamma \left(n,kxa\right)$ is an incomplete gamma function of the first kind^18^ (see^19^). This solution is useful because the analysis sometimes requires the explicit time-dependent solution of ${P}_{n}(a)$.

#### Longevity, cancer risk, and age-incidence relation

We begin with the total mortality due to cancer and the mean longevity. The probability that a newly born individual survives until age $a$ is given as follows:

3 $$l\left(a\right)=\left(1-{P}_{n}\left(a\right)\right){e}^{-ua},$$

where $u$ is the instantaneous rate of mortality due to factors other than cancer. We call it "noncancerous mortality". Here, we assume that it is a constant: hence, the survivorship considering non-cancer death only would simply be an exponential function of age. This assumption greatly simplifies the mathematical analysis (see below). For simplicity, we here neglect a high mortality at birth. The exponential decline of $l(a)$ is called "Type 2 survivorship" in population ecology and is observed among some animal species^20^. Note that ${P}_{n}(a)$ depends on $x$, the error rate of genomic replication, and hence the survivorship function $l(a)$ also depends on $x$.

Below, we show how the environmental improvement causes changes in the mean longevity, total fraction of individuals that eventually die of cancer, and age-dependent cancer incidence. Since these changes occur within several generations, we can regard $x$ as a fixed constant. In contrast, if we consider the changes over numerous generations, evolution may occur and change the genomic error rate $x$. The outcomes of the evolutionary changes in $x$ will be discussed in the next section.

##### Total mortality due to cancer

We consider the fraction of individuals who eventually die of cancer, and we call it as "total mortality of cancer" and denoted by $${M}_{C}$$. In contrast, the fraction of individuals who die of factors other than cancer is denoted by as $${M}_{N}$$ ($$=1-{M}_{C})$$. The total mortality caused by the factors other than cancer is

4a $${M}_{N}={\int }_{0}^{\infty }u\left(1-{P}_{n}\left(a\right)\right){e}^{-ua}da,$$

and the total mortality due to cancer, or total cancerous mortality, is

4b $${M}_{C}=1-{M}_{N}=\prod_{j=0}^{n-1}\frac{{k}_{j}x}{u+{k}_{j}x}.$$

**Figure 2:**
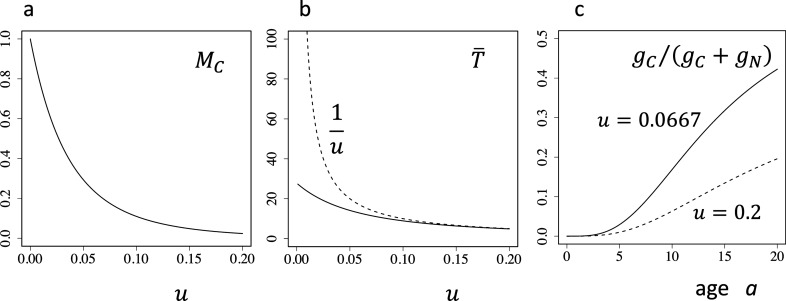
Response of the population to the improved environment. (**a**) Total mortality due to cancer ${M}_{C}$. The horizontal axis represents $u$, the mortality due to processes other than cancer. As the environmental condition is improved, $u$ becomes smaller, and ${M}_{C}$ increases. (**b**) Mean longevity $\stackrel{-}{T}$. The horizontal axis is $u$. The solid curve is the mean longevity. The broken curve is $1/u$. (**c**) Fraction of mortality due to cancer at each age $a$, ${g}_{C}\left(a\right)/\left({g}_{C}\left(a\right)+{g}_{N}\left(a\right)\right)$. The two curves are for different values of $u$, as indicated by the label next to each curve. As the environment is improved, $u$ becomes smaller, and the fraction of cancer mortality at each age increases. Note that the absolute mortality due to cancer ${g}_{C}\left(a\right)$ is independent of $u$. Parameters are: $k=1.8$, $x=0.1$, and $n=5$.

See SI Appendix A for the derivation. Equation (4b) shows that ${M}_{C}$ decreases with increasing $u$, as shown in Fig. 2a. As the environment is improved, $u$ becomes smaller, and the fraction of cancer among total mortality increases. In the absence of other mortality factors ($u=0$), ${M}_{C}$ becomes 1. This is the direct dependence of the total cancer mortality on the improved environment.

##### Mean longevity

The mean longevity is denoted by $\stackrel{-}{T}$, and is given as follows:

5 $$\stackrel{-}{T}={\int }_{0}^{\infty }a\left[-\frac{\partial }{\partial a}l\left(a\right)\right]da=\frac{1}{u}\left(1-\prod_{j=0}^{n-1}\frac{{k}_{j}x}{u+{k}_{j}x}\right).$$

See SI Appendix A for the derivation. Since $1/u$ is the mean longevity in the absence of cancer, the last expression of Eq. (5) indicates that the mean longevity is reduced by cancer, and the magnitude of the reduction is explicitly calculated. Note that the negative term within the parentheses in the last expression of Eq. (5) is equal to ${M}_{C}$, given by Eq. (4b). Equation (5) indicates ${M}_{N}=u\stackrel{-}{T}$, which implies that the total noncancerous mortality is equal to the per-year noncancerous mortality multiplied by mean longevity.

When the environment becomes benign, $u$ becomes smaller and the longevity Eq. (5) becomes larger. Figure 2b shows how $\stackrel{-}{T}$ depends on $u$. As mortality $u$ decreases, the terms within the parentheses decrease, but the effect of the factor $1/u$ is stronger, and the longevity $\stackrel{-}{T}$ increases as the environment is improved. In Appendix A, we proved that $\stackrel{-}{T}$ decreases with $u$ for all $n=1$, 2, 3, …

##### Age-dependent mortality due to cancer

The age-dependent instantaneous mortality due to cancer is given as follows:

6 $${g}_{C}\left(a\right)=\frac{\partial }{\partial a}{P}_{n}\left(a\right)/\left(1-{P}_{n}\left(a\right)\right)={c}_{n-1}{P}_{n-1}\left(a\right)/\left(1-{P}_{n}\left(a\right)\right),$$

as a function of age $a$ (see SI Appendix A for the derivation). This is a fraction of individuals who developed cancer within a short time interval among those who survived until age $a$. This expression requires the explicit solution of ${P}_{n-1}\left(a\right)$, which needs to be calculated numerically from Eq. (1), in general cases. However, when the transition rates between states are equal, we have the explicit solution as Eq. (2), and we can derive the following:

7 $$\begin{aligned}{g}_{C}\left(a\right)&=kx\frac{{\left(kx\right)}^{n-1}{a}^{n-1}}{\left(n-1\right)!}{e}^{-kxa}/\left(1-\frac{1}{\left(n-1\right)!}\gamma \left(n,kxa\right)\right)\\&={\left(kx\right)}^{n}{a}^{n-1}{e}^{-kxa}/\left(\left(n-1\right)!-\gamma \left(n,kxa\right)\right).\end{aligned}$$

Note that Eq. (7) does not contain $u$ because the instantaneous mortality calculated for each age is independent of $u$.

##### Age-dependent relative fraction of cancer mortality

The corresponding instantaneous mortality due to processes other than cancer is ${g}_{N}\left(a\right)=u$. The relative fraction of cancer mortality among the total deaths within a unit time is ${g}_{C}\left(a\right)/\left({g}_{C}\left(a\right)+{g}_{N}\left(a\right)\right)$. A smaller $u$ increases the quantity.

To exactly determine this quantity, we need to know the explicit solution of ${P}_{n}\left(a\right)$. When all transition rates are equal, we can use Eq. (7) with ${g}_{N}\left(a\right)=u$. Figure 2c illustrates this case. The fraction of cancer mortality among all mortality factors is found to increase with age $a$. We show the curves for two different values of $u$.

In short, the improvement of the living conditions (smaller $$u)$$ enhances the total cancerous mortality $${M}_{C}$$. It does not change the age-specific cancer incidence $${g}_{C}\left(a\right)$$, but enhances the relative fraction of age-specific mortality due to cancer $${g}_{C}\left(a\right)/\left({g}_{C}\left(a\right)+{g}_{N}\left(a\right)\right)$$.

### Evolutionary adaptation and the indirect effect of longevity

In the analysis discussed in the last section, we treated genomic error rate $x$ as a constant, not a quantity to evolve. Within a few generations, the evolutionary changes in traits would be rather small. For the case of dogs with mean generation time of five years, the changes in 10–30 years can be interpreted as the response of the population adapted to the original environment to the new environmental situations. However, after hundreds of generations, phenotypic traits that receive selection would experience changes adapted to the new environment. For the case of dogs, this may require the time about 1000 years or longer. After many generations, those individuals who achieve a higher reproductive success than others tend to increase their share and replace the old types.

After many generations, what remains in the population is the phenotype that achieves the largest lifetime reproductive success or fitness. This is the basic logic underlying the use of optimization and game in evolutionary ecology. These theoretical tools have been very successful in understanding many traits of organisms, including behavior, morphology, and life history. Herein, we considered the genomic error rate as a quantity that can evolve under natural selection. We calculate the fitness for different values of $x$ and postulate that the value of $x$ that achieves the maximum fitness is the one adopted by the organisms we observed in the current world. The population genetics theory concluded that the evolution by natural selection can be understood in terms of the lifetime reproductive success of an individual, or the fitness^21^. Even if the evolutionary change causes population or ecosystem outcomes, the unit of selection is an individual, not population or species^22^.

#### Fitness

Reducing genomic replication error $x$ is adaptive because it reduces the risk of cancer. However, it requires investment of resources to the machinery of genome replication or increasing the degree of surveillance activities such as the immune system. In the evolutionary biology of mutation rates, the observed genomic error rate can be understood as the balance of benefit of reducing error, and a high cost to further reduce the error rate^23,24^. We here assume that the attempts to reduce $x$ further are accompanied by a cost. We considered that the fertility decreases by investing more in machinery, reducing the chance of mistakes. In particular, we considered the cost as a power function ${f}_{0}/{x}^{q}$, which decreases with the error rate $x$. If we were to assume $x$ to be very small, the cost would be very large and prevent the realization of zero chance of error. Here, we assume the following function of the fertility:

8 $$m\left(a,x\right)={m}_{0}\left(a\right)-\frac{{f}_{0}}{{x}^{q}}.$$

In the absence of this additional cost, fertility is given as ${m}_{0}\left(a\right)$, which is small for age less than the maturity age, increases to the maximum value, and remains the same until the individual becomes aged. It may decrease for a very large age, however, under field condition, where this species has been evolved, not many individuals can survive to the age exhibiting a decline in fertility.

The expected number of offspring to be produced in the lifetime, or the fitness, is given as

9 $$F\left(x\right)={\int }_{0}^{\infty }l\left(a\right)m\left(a,x\right)da={\int }_{0}^{\infty }\left({m}_{0}\left(a\right)-\frac{{f}_{0}}{{x}^{q}} \right)\left(1-{P}_{n}\left(a\right)\right){e}^{-ua}da.$$

${P}_{n}(a)$ is given by Eq. (2b), which is dependent on $x$, because ${c}_{i}={k}_{i}x$.

When the fertility is independent of age, ${m}_{0}\left(a\right)$ is a constant ${m}_{0}$, and we can solve the fitness function as follows:

10 $$F\left(x\right)=\left({m}_{0}-\frac{{f}_{0}}{{x}^{q}} \right)\frac{1}{u}\left(1-\prod_{j=0}^{n-1}\frac{{k}_{j}x}{u+{k}_{j}x}\right).$$

See SI Appendix A for the derivation. We adopted the simplified assumption ${m}_{0}\left(a\right)={m}_{0}$ in the standard case in this study. We later studied the case of age dependence of ${m}_{0}\left(a\right)$.

If the chance of introducing mistake during DNA replication is reduced, survivorship $l(a)$ increases. However, reducing mistakes in replication effort would reduce the rate of replication and require a higher energetic cost, which is represented as the reduction of fertility. As a compromise between these two effects, there exists an intermediate optimal rate of mistake $x$ that maximizes the fitness given by Eq. (9). We assume that, after many generations, this optimal $x$ is realized.

#### Direct and indirect dependences

Herein, we discuss the results for the simple cases in which all the transition rates between states are the same and the fertility is independent of age. Later we describe the results when these assumptions are modified.

**Figure 3:**
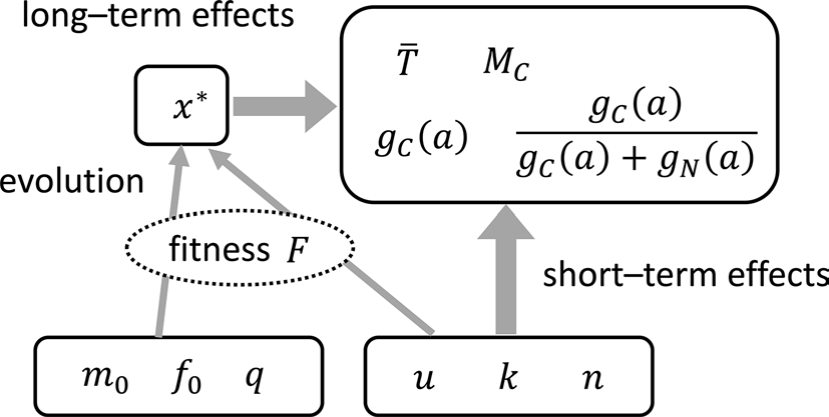
Schematic of how different parameters affect the quantities in the model.

How different quantities in the model affect others is shown in Fig. 3. There are six parameters, three of which (${m}_{0}$, ${f}_{0}$, and $q$) are in the formula of fitness, whereas the other three ($u$, $k$, and $n$) are included in the dynamics of cancer progression. The quantities we are concerned are the mean longevity $\stackrel{-}{T}$, total cancerous mortality ${M}_{C}$, age-dependent cancer mortality ${g}_{C}\left(a\right)$, and the fraction of cancer mortality among total deaths for each age ${g}_{C}\left(a\right)/\left({g}_{C}\left(a\right)+{g}_{N}\left(a\right)\right)$. These statistics depend on the genomic error rate $x$ as well as the parameters included in the cancer progression formula. Note that these statistics depend on $u$, $k$, and $n$ in two ways: they depend directly on these parameters, and they also depend on ${x}^{*}$ (the evolutionarily stable value (ESS) of $x$), which depends on $u$, $k$, and $n$ (see Fig. 3).

#### Evolutionarily stable life history

**Figure 4:**
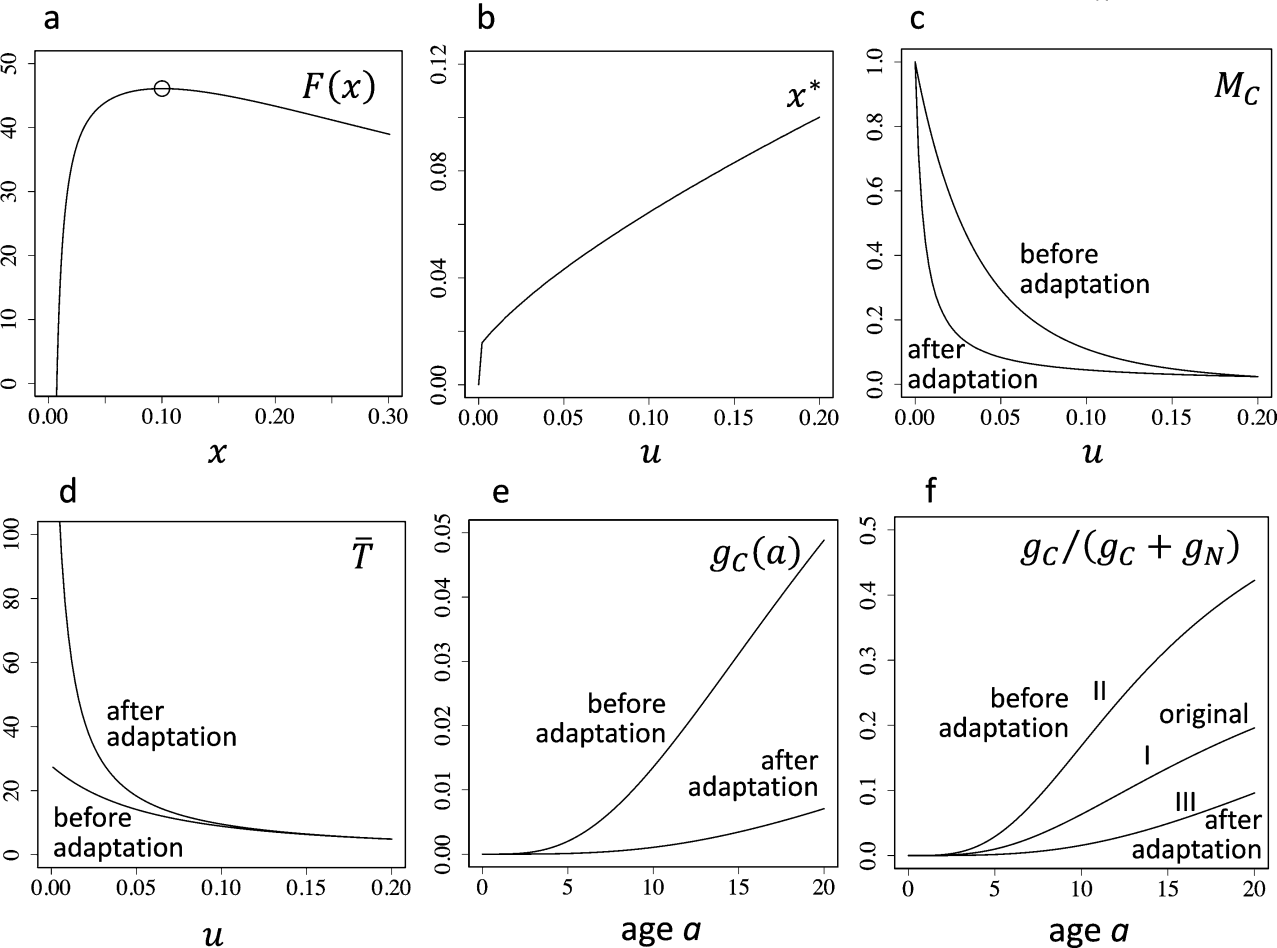
Population response when the genomic error rate can evolve to the ESS. (**a**) Fitness for different genomic error rates. It attains the maximum when $x=0.1$, which is the evolutionarily stable value. The other parameters are $u=0.2$, $k=1.8$, ${m}_{0}=10$, ${f}_{0}=0.044$, $q=1.1$, and $n=5$. (**b**) The evolutionarily stable value of genomic error rate ${x}^{*}$. The horizontal axis represents $u$, noncancerous mortality. The parameters are the same as in (**a**) unless specified otherwise. (**c**) Total cancerous mortality ${M}_{C}$. The curve labeled as "before adaptation" indicates ${M}_{C}$ if the genomic error rate is fixed: $x=0.1$ (the ESS value under $u=0.2$), which is the same as that in Fig. 2a. The curve labeled as "after adaptation" indicates ${M}_{C}$ if the genomic error rate $x$ is equal to the ESS value corresponding to $u$ in the horizontal axis (shown in Fig. 4b). Note that the two curves cross each other at $u=0.2$. (**d**) Mean longevity $\stackrel{-}{T}$. The curve labeled as "before adaption" is $\stackrel{-}{T}$ when $x=0.1$, which is the same as that in Fig. 2b. The curve labeled as "after adaptation" is $\stackrel{-}{T}$ when $x$ is the ESS value corresponding to $u$, given in the horizontal axis. (**e**) Age-specific mortality due to cancer ${g}_{C}\left(a\right)$. The curve labeled as "before adaptation" is the one when $u=0.2$ and $x=0.1$. When the noncancerous mortality is reduced to $u=0.0667$, the curve remains the same. The curve labeled as "after adaptation" is the one for $x=0.0507$, which is the ESS value under $u=0.0667$. (**f**) The relative fraction of cancer mortality at each age $a$, ${g}_{C}\left(a\right)/\left({g}_{C}\left(a\right)+{g}_{N}\left(a\right)\right)$. The curve labeled as I is the one in the original population, in which $u=0.2$ and $x=0.1$ (the ESS value). The curve labeled as II and "before adaptation" is the one when the noncancerous mortality is reduced to $u=0.0667$, without changing $x$. The curve labeled as III and "after adaptation" is the one in which the genomic error rate evolves to the ESS value $x=0.0507$ under $u=0.0667$. The curve III is lower than the other curves. Parameters are the same as in (**a**), unless specified otherwise.

The fitness as a function of $x$, the error rate of genome replication, is shown in Fig. 4a. There exists an intermediate level of $x$ that maximizes the fitness given by Eq. (10). We denote this by ${x}^{*}$.

##### Genomic error rate: $x$

In evolution, the genomic error rate responds to changes in non-cancerous mortality $u$. If $u$ becomes smaller, the longevity increases, and the fraction of mortality by cancer increases. Thus, the reduction of cancer risk by reducing the genomic error rate $x$ becomes more profitable if $u$ is large. The ESS genomic error rate ${x}^{*}$ for different $u$, is shown in Fig. 4b. The change in $x$ subsequently modifies other parameters and causes indirect effects via evolution (see Fig. 3). The evolutionary outcomes reflect both direct effects and the indirect effects shown below.

##### Total cancerous mortality: ${M}_{C}$

The two curves in Fig. 4c illustrate ${M}_{C}$, the fraction of cancerous mortality among the total mortality. The horizontal axis represents the noncancerous mortality $u$ in the new environment. The curve labeled as "before adaptation" indicates ${M}_{C}$ without evolutionary adjustment of $x$, which is maintained as $x=0.1$ (the ESS under $u=0.2$). This curve is the same as that shown in Fig. 2a. The other curve labeled as "after adaptation" is the total cancerous mortality ${M}_{C}$ after the evolutionary adjustment of $x$. The second curve is considerably lower than the first one for $u<0.2$.

In the population originally at the ESS under $u=0.2$, the total cancerous mortality ${M}_{C}$ was 0.0238. If the population was suddenly exposed to a smaller noncancerous mortality $u=0.0667$, ${M}_{C}$ became 8.7 times larger: ${M}_{C}=0.2068$. If this new environment lasts for many generations, the value of $x$ evolves to the ESS value, and the cancerous mortality is reduced to ${M}_{C}=0.0644$, which is considerably smaller than the one before adaptation, ${M}_{C}=0.2068$. However, ${M}_{C}=0.0644$ is still larger than the original value ${M}_{C}=0.0238$. This is the outcome in which both the direct change and the indirect evolutionary change are combined, implying that the former was stronger than the latter.

##### Mean longevity: $\stackrel{-}{T}$

The two curves in Fig. 4d illustrate the mean longevity caused by the decrease in $u$. The original value of noncancerous mortality was $u=0.2$ (the mean longevity was about 5 years). The curve labeled as "before adaptation" indicates the direct change of $\stackrel{-}{T}$ in response to the environmental change to a reduced value of $u$, indicated in the horizontal axis when $x$ is maintained at the ESS level under $u=0.2$. The other curve, labeled as "after adaptation" indicates the mean longevity after the adaptive evolution of $x$ in response to the reduced $u$ (see Fig. 4b). The two curves differ significantly, implying that the evolutionary response of the genomic error rate $x$ improved the longevity, especially for a small $u$.

##### Age-dependent instantaneous mortality due to cancer: ${g}_{C}\left(a\right)$

The two curves in Fig. 4e illustrate the age-dependent mortality due to cancer. The curve labeled as "before adaptation" was calculated using the genomic error rate $x=0.1$ (the ESS under $u=0.2$). If the environment became improved, $u$ became smaller, but this curve ${g}_{C}\left(a\right)$ remained unchanged, as it is independent of $u$. The curve labeled as "after adaptation" is the one when the genomic error rate is adjusted by evolution to be a smaller value: $x=0.0507$, which is the ESS under $u=0.0667$. We can see that cancer mortality at every age was reduced by the evolutionary adaptation of $x$.

##### Age-dependent fraction of cancerous mortality: ${g}_{C}\left(a\right)/\left({g}_{C}\left(a\right)+{g}_{N}\left(a\right)\right)$

Three curves are shown in Fig. 4f. The curve labeled as I is the value in the original environment where noncancerous mortality was high ($u=0.2$), and the genomic error rate was the ESS in that environment ($x=0.1$). The curve labeled as II is the one in the improved environment with smaller noncancerous mortality ($u=0.0667$), where the genomic error rate was the same as in the original environment ($x=0.1$). The curve labeled as III is the one after the genomic error rate is adjusted to the ESS value in the improved environment ($x=0.0507$). The age-specific fraction of cancer mortality was found to be lower than that in the original environment. This implies that an animal adapted in a benign environment should have less chances of developing cancer than the one adapted in a severe environment.

## Discussion

Cancer occurs as an outcome of accumulated mistakes in the genome and/or epigenome. The cancer incidence data have been analyzed in terms of the multi-step model (or multistage model;^3^), which postulates that the individuals are born with step 0, the random transition occurs to increase the step number by one, and the individual would develop malignant cancer when the step number reaches $n$ (Fig. 1). The transition rates in this model represent the rate of somatic mutations or other somatic deformation of key genes controlling the tumor surveillance and suppression. These are caused by mistakes in somatic genomic replication (mistakes in DNA or epigenetic marks); however, the error rate can be reduced, if the host invests resources into a machinery to perform accurate genomic replication and to detect cancerous cells and remove them, which would slow down the accumulation of mutation events. The rate of accumulation of adult stem cells during the human lifetime is tissue-specific^25^.

We could explicitly calculate the total mortality due to cancer ${M}_{C}$ as shown in Eq. (4b). We also derived a formula for the mean longevity $\stackrel{-}{T}=\left(1/u\right)\left(1-{M}_{C}\right)$. Since $1/u$ is the mean longevity in the absence of cancer, the cancer mortality reduces the mean longevity exactly by the fraction of ${M}_{C}$.

The age-specific mortality due to cancer ${g}_{C}\left(a\right)$ is given by Eq. (6) which requires an explicit expression of age-dependent solution. In many wild populations, the individuals with cancer constitute a small fraction of the total population. If so, we have the following approximation:

11 $${g}_{C}\left(a\right)\approx \left(\prod_{j=0}^{n-1}{c}_{j}/\left(n-1\right)!\right)\bullet {a}^{n-1},$$

where ${c}_{j}={k}_{j}x$ (derived in SI Appendix A). Note that the accumulated incidence of cancer ${\int }_{0}^{a}{g}_{C}\left(a^{\prime}\right)da^{\prime}$ is proportional to ${a}^{n}$, which is often used to obtain the step number $n$ from the data ^3^. Under this approximation the relative fraction of age-dependent mortality is ${g}_{C}\left(a\right)/\left({g}_{C}\left(a\right)+{g}_{N}\left(a\right)\right)\approx \left(1/u\right){g}_{C}\left(a\right)$, which increases with the longevity.

### Direct and indirect effects

In this paper, we focus on how the reduction of noncancerous mortality enhances the importance of cancer as a mortality factor. Companion animals in contemporary society live in an environment with sufficient food supply, protection against danger, and medical treatments, where the mortality due to factors other than cancer (e.g., infectious diseases, physical accidents, and food shortage), denoted by $u$, is reduced. They experience a direct change in $u$ but not the evolutionary change in the genomic error rate $x$.

One might think that the enhanced cancerous mortality of companion animals might be caused by consumption of unnatural food, hypokinesia, obesity, etc. We do not reject their effect. However, the key messages of the current paper are to clearly present that observed enhancement of the cancer mortality of companion animals can be caused by the reduction of non-cancerous mortality by the improvement of the environment. Even if there is no enhancement of the rate of tumor promotion steps, the cancerous mortality is greatly enhanced simply by the improved environment.

After numerous generations, the genomic error rate $x$ should evolve in response to the improved environment. The evolutionary changes explain why long-lived species (e.g. elephants) have evolved mechanisms to reduce the risk of cancer^7–9^. This idea can be supported by considering both the direct effects of reduced $u$ and the indirect effects via the evolutionary reduction of $x$.

The $n$-step model for cancer predicts that, when the noncancerous mortality $u$ decreases, the age-dependent mortality due to cancer ${g}_{C}\left(a\right)$ remains the same, and the relative importance of cancer as a mortality factor at each age increases (Fig. 2c), leading to the increase in the total mortality due to cancer ${M}_{C}$ (Fig. 2a). If the cancer mortality is small, the relative importance of cancer in age-specific mortality ${g}_{C}\left(a\right)/\left({g}_{C}\left(a\right)+{g}_{N}\left(a\right)\right)$ should increase with the longevity in proportion to $1/u$. Interestingly, the total cancerous mortality ${M}_{C}$ should increase in proportion to ${\left(1/u\right)}^{n}$, which has a stronger dependence than the importance of age-specific mortality if the step number $n$ is large (see Eq. (4b) when ${k}_{j}\ll u$). This suggests that companion animals are expected to develop solid cancers (with a large $n$) more than leukemia (with a small $n$; see SI Appendix B for further argument on the dependence on step number $n$).

After many generations in the environment with a small $u$, the genomic error rate $x$ evolves to a smaller value (see Fig. 4b), which reduces the total cancerous mortality ${M}_{c}$. Hence, the indirect effect operates in the direction opposite to the direct effect. The sum of the direct and indirect effects of $u$ on ${M}_{c}$ depends on the magnitude by which ${x}^{*}$ changes in response to $u$. In the case assessed in this study, ${M}_{c}$ was small (0.0238) in the original environment with $u=0.2$; ${M}_{c}$ became considerably larger (0.2068) after the environment was improved (u = 0.0667). However, after evolutionary adjustment of $x$, ${M}_{c}$ became small again (0.0644), which was still larger than the original value, indicating that the direct effect was stronger than the indirect one.

The indirect effect of enhanced longevity (larger $1/u$) would make ${x}^{*}$, and thus the age-specific cancer mortality ${g}_{C}\left(a\right)$, smaller. Since the direct effect does not change ${g}_{C}\left(a\right)$, the net effect of evolution would always reduce the age-specific cancer mortality (Fig. 4e).

The indirect effect of enhanced longevity reduces the relative importance of cancer to age-specific mortality ${g}_{C}\left(a\right)/\left({g}_{C}\left(a\right)+{g}_{N}\left(a\right)\right)$. However, this is in the direction opposite to the direct dependence. Hence, the evolutionary change, which is the combined effects, depends on the magnitude of change in ${x}^{*}$.

### Extensions of the model

We explained the model only for the standard situation, in which the step number is $n=5$, the fertility is independent of age, and the transition rates between step numbers are equal, and the cost function for error reduction has a power $q=1.1$. We also analyzed the cases where these assumptions were modified. The analyses are explained in SI Appendix B. Below we summarize the main conclusions.

#### Shape of cost function for reducing genomic error rate

The magnitude of evolutionary response of the genomic error rate ${x}^{*}$ should depend on the shape of the cost function ${f}_{0}/{x}^{q}$. If $q$ is large, the cost is enhanced sharply as the error rate is reduced, and the ESS $x$ would not change considerably when $u$ becomes smaller. In contrast, if $q$ is smaller, the response of ${x}^{*}$ to $u$ is stronger because the cost accompanied by reduced $x$ is smaller. In SI Appendix B, we provide the analysis results for the cases with three different values: $q=0.5$, $q=1.1$, and $q=2$. If the noncancerous mortality is $u=0.0667$, the ESS is ${x}^{*}=0.0424$ for $q=0.5$; ${x}^{*}=0.0507$ for $q=1.1$; and ${x}^{*}=0.0599$ for $q=2$ (see Table S1). Thus, $x$ responds more sensitively to the change in $u$ when $q$ is small than when it is large (see Fig. S1).

The reduction of the total cancerous mortality ${M}_{C}$ by the evolutionary adaptation of $x$ is stronger for smaller $q$. For example, for $q=0.5$, under $u=0.0667$ the evolution renders cancerous mortality to become as low as ${M}_{C}=0.0433$, which is lower than the corresponding value ${M}_{C}=0.0644$ for $q=1.1$. However, even this value is greater than ${M}_{C}=0.0238$, the value in the original environment with high mortality ($u=0.2$; see Table S1).

These analyses suggest that cancerous mortality is reduced by the evolutionary adjustment of genomic error rate, and the magnitude of reduction is stronger for a smaller $q$. However, the reduced fraction of total cancerous mortality is still larger than the value in the original population having a high noncancerous mortality rate (see SI Appendix B for more details).

#### Smaller step number

The step number is about 5 or 6 for many solid tumors, but it is smaller for leukemia. It may depend on the number of mutations required before the cancer starts, although they are not exactly the same^5^. If the step number was smaller, the model would predict cancer occurs at younger ages. Irrespective of $n$, we observed the enhancement of the total cancer mortality ${M}_{C}$ by the improved environment (i.e., smaller $u$) and the reduction of ${M}_{C}$ after evolutionary adjustment. However, both of these reactions were weaker for small $n$ than for large $n$ (see Fig. S2). The procedure of comparison and analyses are explained in SI Appendix B.

From these results, we conclude that the enhanced cancer risk caused by the improved environment is more significant for solid tumors than for leukemia.

#### The transition rate becomes faster in an accelerating manner

We may consider the case in which the transition rates from one step number to the next become faster as the step number progresses. There are several reasons for this assumption. Cancer cells initially form a small colony; however, the colony becomes larger as it accumulates mutations in tumor suppressors and oncogenes, resulting in faster cell division (or slower cell death). Progression at later stages may occur when at least one of the many cells in the colony experiences mutations, which would be faster than receiving one mutation in a small number of stem cells during progression in the earlier stages. After the cells acquire the ability of angiogenesis, the colony size becomes considerably larger, as they can attract the vascular system that supplies nutrition to tumor cells. In addition, some cancers are known to start from the mutations that cause genomic instability, for example chromosomal instability or microsatellite instability in colon cancer^1,2^, which enhances the speed of changes in the later steps.

The analysis in SI Appendix B suggests us that the acceleration of the transition speeds should act in a manner similar to the reduced step number (see Fig. S3).

#### Age-dependent fertility

We performed the analysis when the fertility is not a constant but is dependent on age. In particular, in SI Appendix B, we show the analysis of the case in which ${m}_{0}\left(a\right)$ is zero both for individuals younger than one year and for those older than 10 years, and takes the maximum value from two years to six years of age. This pattern was chosen to imitate the fertility of dogs.

According to the data shown in SI Appendix B, the evolutionary response was noticeable for the genomic error rate for the improved environment (smaller $u$), but the magnitude of the response was very small (see Fig. S4). This is because the natural selection working to remove cancerous mortality is weak, as most cancerous mortality occurs in the old individuals when the fertility is zero. We also calculated the total cancerous mortality ${M}_{C}$, which after adaptation was smaller than before adaptation. However, the magnitude of the reduction was very small.

We can conclude that the evolutionary response of the population to the improved environment is small and the cancerous mortality remains high even after the evolution of ${x}^{*}$, probably because natural selection acting to reduce cancer risk is weak.

The theory for companion animals in this study may be applicable to humans. We could eliminate many mortality factors that have been very serious problems in history. Because of the improvements in living conditions, including improvement in food supply, nutrition, shelter, hygiene, and health care, the mean longevity of humans has been increased considerably, which is ubiquitous in all areas of the world^26^. Similar to pet animals, we human beings have a higher rate of cancer mortality among all the mortality factors, especially those occurring in old age. However, the formalism in the current paper regard the lifetime reproductive success as the fitness, which may not be the case for human beings considering female menopause^27^.

Noncancerous diseases such as neurological disorders have been considered to be caused by somatic deformations of the genome^28,29^. The present model may also be applicable to these diseases.

## Supplementary information

Supplementary Information

## Data Availability

Data sharing is not applicable to this article as no datasets were generated or analyzed during this study.
