# Supplementary material for: Enhanced risk of cancer in companion animals as a response to the longevity
Source: Sci Rep. 2020 Nov 11;10:19508. doi: 10.1038/s41598-020-75684-4 (PMC7658259; doi:10.1038/s41598-020-75684-4)
Supplement: Supplementary file 1 — Supplementary Information [file 41598_2020_75684_MOESM1_ESM.pdf]

SUPPLEMENTARY INFORMATION

(Appendices A and B)

Enhanced risk of cancer in companion animals as a response to the longevity

Moeko Tanaka, Sachi Yamaguchi, and Yoh Iwasa

Appendix A

Analysis of the n-step model for cancer

A.1 Laplace transforms

We start with equation (1), the differential equations for $P_i(a)$ $(i = 0, 1, 2, \dots n)$ and the initial condition: $P_0(0) = 1$ and $P_1(0) = P_2(0) = \dots P_n(0) = 0$. Because differential equations are linear, Laplace transforms are useful. We define

$$\hat{P}_i(\sigma) = \int_0^\infty P_i(a)e^{-\sigma a}da. \qquad \text{for } i = 0, 1, 2, \dots n. \qquad \text{(A.1)}$$

Then equation (1) becomes

$$\sigma\hat{P}_0(\sigma) - 1 = -c_0\hat{P}_0(\sigma) \ , \qquad \text{(A.2a)}$$

$$\sigma\hat{P}_i(\sigma) = c_{i-1}\hat{P}_{i-1}(\sigma) - c_i\hat{P}_i(\sigma), \qquad \text{for } i = 1, 2, \dots n-1 \qquad \text{(A.2b)}$$

$$\sigma\hat{P}_n(\sigma) = c_{n-1}\hat{P}_{n-1}(\sigma) \quad . \qquad \text{(A.2c)}$$

From these, we have

$$\hat{P}_i(\sigma) = \frac{1}{\sigma+c_i}\prod_{j=0}^{i-1}\frac{c_j}{\sigma+c_j} \quad , \qquad \text{for } i = 0, 1, \dots n-1 \qquad \text{(A.3a)}$$

and $$\hat{P}_n(\sigma) = \frac{1}{\sigma}\prod_{j=0}^{n-1}\frac{c_j}{\sigma+c_j} \quad . \qquad \text{(A.3b)}$$

The general property $P_n(\infty) = lim_{\sigma\to 0}\{\sigma\hat{P}_n(\sigma)\} = 1$ implies that all individuals should develop cancer if not killed by noncancer mortality factors.

A.2 Total mortality due to cancer and to other processes

Mortality due to noncancer processes is calculated as $l(a)$, the fraction of people who can survive until age a multiplied by the instantaneous rate of noncancerous mortality u then integrated over all ages: $M_N = \int_0^\infty ul(a)da$. Using equation (3), this can be rewritten as follows:

$$M_N = \int_0^\infty u\big(1 - P_n(a)\big)e^{-ua}da = 1 - u\hat{P}_n(u) = 1 - \prod_{j=0}^{n-1} \frac{c_j}{u+c_j}, \qquad \text{(A.4a)}$$

where we adopted the Laplace transform $\hat{P}_n(\sigma)$.

Then the mortality due to cancer, namely the fraction of individuals who die of cancer is

$$M_C = \prod_{j=0}^{n-1} \frac{c_j}{u+c_j} \ . \qquad \text{(A.4b)}$$

If we replace $c_j = k_j x$ for $j = 0, 2, \ldots, n-1$, then equations (A.4a) and (A.4b) become equations (4a) and (4b) in the text.

A.3. Mean longevity

Note that $(-1)\frac{\partial}{\partial a} l(a, x)$ is the fraction of individuals that die from age a within one time unit (Here to make the dependence on x clear, we use $l(a, x)$ instead of $l(a)$). Then the mean longevity is

$$\bar{T} = \int_0^\infty a\left[-\frac{\partial}{\partial a} l(a, x)\right] da = \int_0^\infty l(a, x)da \qquad . \qquad \text{(A.5)}$$

The last term is rewritten from the middle term through the integration by parts and the boundary conditions: $\lim_{a\to\infty} al(a, x) = 0$ and $l(0, x) = 1$. From equation (A.5), we have

$$\bar{T} = \int_0^\infty \big(1 - P_n(a)\big)e^{-ua}da = \frac{1}{u}\left(1 - \prod_{j=0}^{n-1} \frac{c_j}{u+c_j}\right) \quad . \qquad \text{(A.6)}$$

Note that this mean longevity is shorter than $1/u$, which is the mean longevity in the absence of cancer.

We can prove that the mean longevity $\bar{T}$ given by equation (A.6) is a decreasing function of u for $n = 1, 2, 3, \ldots.$, based on the induction with respect to n. To indicate the dependence on n explicitly, we write the mean longevity as $\bar{T}_n$, which is given by equation (A.6).

When $n = 1$, we have

$$\bar{T}_1 = \frac{1}{u}\left(1 - \frac{c_0}{u+c_0}\right) = \frac{1}{u} \cdot \frac{u}{u+c_0} = \frac{1}{u+c_0} \quad , \qquad \text{(A.7)}$$

which is a monotonically decreasing function of u.

Suppose that $\bar{T}_n$ is a monotonically decreasing function of u. Consider the equation with $n + 1$:

$$\bar{T}_{n+1} = \frac{1}{u}\left(1 - \prod_{j=0}^{n} \frac{c_j}{u+c_j}\right) = \frac{1}{u}\left(1 - \frac{c_n}{u+c_n} \prod_{j=0}^{n-1} \frac{c_j}{u+c_j}\right)$$

$$= \frac{1}{u}\left(1 - \left(1 - \frac{u}{u+c_n}\right) \prod_{j=0}^{n-1} \frac{c_j}{u+c_j}\right) = \bar{T}_n + \frac{1}{u+c_n} \prod_{j=0}^{n-1} \frac{c_j}{u+c_j} \ . \qquad \text{(A.8)}$$

The last expression is a sum of two decreasing function of u. Hence, we can conclude that $\bar{T}_{n+1}$ is a monotonically decreasing function of u.

Taken together we can conclude that $\bar{T}_n$ is a monotonically decreasing function of u for all $n = 1, 2, 3, \ldots$

A.4. Age-specific mortality due to cancer

Now we consider the mortality rate for individuals of age a. We consider many individuals who survive until age a. Then, ask what is the fraction of individuals that may die in a year (or one time unit)? Since the survivorship until age a is $l(a, x)$, the number of individuals that die in unit time is $(-1)\frac{\partial}{\partial a} l(a, x)$, which is the fraction among those born

in age 0. The fraction of this number among those survives until age a is $(-1)\frac{\partial l}{\partial a}/l(a,x)$, which is the instantaneous mortality at age a. From equation (3), we have $l(a,x) = \big(1-P_n(a)\big)e^{ua}$, we have

$$(-1)\frac{\partial l}{\partial a}/l(a,x) = (-1)\frac{\partial\big(1-P_n(a)\big)}{\partial a}/\big(1-P_n(a)\big) + u \quad . \tag{A.9}$$

The first term on the right-hand side is the instantaneous mortality due to cancer, which we denote by $g_C(a)$. The second term is the instantaneous mortality due to processes other than cancer (e.g., food shortage, physical damage, infectious diseases, and all the other processes that kill an individual).

$$g_C(a) = \frac{c_{n-1}P_{n-1}(a)}{1-P_n(a)} \quad , \quad g_N(a) = u \quad . \tag{A.10}$$

The first equation is the same as equation (6) in the text. In simple situations, we have an explicit formula for $P_{n-1}(a)$ and $P_n(a)$. For example, when all the transition rates are equal, we have equations (2a) and (2b). Otherwise, we need to calculate them numerically.

The ratio of the first term to the sum of equation (A.10) gives the fraction of cancer as a mortality factor, as follows:

$$\frac{g_C(a)}{g_C(a)+g_N(a)} = \frac{c_{n-1}P_{n-1}(a)}{c_{n-1}P_{n-1}(a)+u\big(1-P_n(a)\big)} \quad . \tag{A.11}$$

<u>*A.4.1 Age-specific cancer risk $g_C(a)$ monotonically increases with age a*</u>

Equation (A.10) indicates that the age-specific cancer risk $g_C(a)$ is proportional to the fraction of surviving individuals that are in state $n-1$, just one step before the cancer incidence state.

From equation (2a), we have

$$P_i(a) = b_i a^i e^{-kxa} \quad , \tag{A.12}$$

where we set $b_i = (kx)^i/i!$. Hence, we have

$$g_C(a) = \frac{kxb_{n-1}a^{n-1}}{b_0+b_1a+..+b_{n-1}a^{n-1}} \quad , \tag{A.13}$$

which is a monotonically increasing function of a. It increases from $g_C(0) = 0$ to the asymptotic value $g_C(\infty) = kx$.

A.4.2. When the age-specific cancer mortality is very small (or small a)

If the fraction cancerous mortality is very small, we have a simple approximate formula equation (11) that provides a useful way to calculate step number from the cancer incidence data. In the model, an individual will certainly die of cancer if it is not killed by noncancerous processes. We hence focus on the behavior of the model with small a. Since $P_0(0) = 1$ and $P_1(0) = P_2(0) = \cdots = P_n(0) = 0$, the age-dependent cancerous mortality, $g_C(a)$, given by equation (A.10) becomes $g_C(a) \approx c_{n-1}P_{n-1}(a)$ in this limit.

The behavior of a function for small a is known from the behavior of the Laplace transform when σ is very large. The Laplace transform of $P_{n-1}(a)$ is given by equation (A.3b), which becomes

$$\hat{P}_{n-1}(\sigma) = \frac{1}{\sigma}\prod_{j=0}^{n-2}\frac{c_j}{\sigma+c_j} \approx \frac{1}{\sigma^n}\prod_{j=0}^{n-2} c_j \quad , \tag{A.14}$$

when $\sigma \gg c_j$ for $j = 0, 1, 2, .., n-2$. Because the Laplace transform of a^{n-1} is $(n-1)!/\sigma^n$, we have

$$P_{n-1}(a) = \frac{\prod_{j=0}^{n-2} c_j}{(n-1)!}a^{n-1} \quad , \tag{A.15}$$

which is equation (11) in text.

Appendix B

We observed the following phenomenon in the standard model: the total cancerous mortality M_C was low in the original population where noncancerous mortality was high ($u = 0.2$). When the animals were suddenly placed in a benign environment with smaller noncancerous mortality ($u = 0.0667$), the total cancerous mortality was enhanced. After many generations, the genomic error rate decreased, and the total cancerous mortality decreased, although the noncancerous mortality remained low ($u = 0.0667$). However, the mitigated total cancer mortality M_C in the new benign environment was still larger than the original value of M_C.

In this appendix, we provide information regarding the extensions of the model in several different directions; we determined whether these results still hold, and whether the responses of the population become stronger or weaker. We examined the following four aspects: (i) the effect of q, which controls the shape of the cost function to reduce the genomic error rate; (ii) the effect of step number n; (iii) whether the transition rate becomes faster as cancer progresses; and (iv) whether the fertility has a peak in an intermediate age and is low for both very young and very old ages.

We focused on their effects on total cancerous mortality. To compare the results with the standard model, we used the following procedure: First, we adjusted the genomic error rate to produce the same value of M_C as in the original environment ($u = 0.2$). Second, we reduced noncancerous mortality to $u = 0.0667$, and elucidated the increase in M_C. Third, we assessed the evolutionary adjustment of the genomic error rate x. To calculate this, we set the original value of the genomic error rate as $x = 0.1$, and we chose the magnitude of cost for reducing the error rate f_0 to make $x^* = 0.1$ the ESS in the original environment with $u = 0.2$. In the new environment with $u = 0.0667$, this value is no longer the ESS. We calculated the ESS value x^* that achieves the maximum fitness

$F(x)$. It should be smaller than the original value. We then calculated the total cancerous mortality M_C. Note that there are three different values of M_C.

B.1 Effect of power q on the cost function for reducing the genomic error rate

The shape of the cost function for reducing the genomic error rate would determine the response of ESS x^* to environmental changes. Power q controls the way the cost increases as x becomes close to zero.

For $q = 0.5$, we adjusted f_0 for the ESS x to be 0.1 when $u = 0.2$, in order to make the total cancerous mortality equal to $M_C = 0.0238$, the latter being the same value as for $q = 1.1$. Then, by using this f_0 and $q = 0.5$, we obtained the ESS x^* as illustrated in Fig. S1 (see the curve labeled as $q = 0.5$). The response of the ESS x^* to the noncancerous mortality u, indicated in the horizontal axis, is stronger than in the case of $q = 1.1$. We also performed a similar calculation for $q = 2.0$. The curve labeled as $q = 2.0$ in Fig. S1 indicates that the evolutionary response of the population to the changed environment is weaker for $q = 2.0$ than for $q = 1.1$.

Then the decrease in the genomic error rate to the ESS value x^* should result in a mitigated cancerous mortality. For example, for $q = 0.5$, the ESS x under $u = 0.0667$ is $x^* = 0.0424$, which leads to the total cancerous mortality $M_C = 0.0433$. We found that the total cancerous mortality after evolutionary adjustment of x is smaller than $M_C = 0.0644$, the latter being the one with the ESS x for $q = 1.1$.

We also performed the same calculation for $q = 2.0$. The results for three different values of q ($q = 0.5$, 1.1, and 2.0) are summarized in Table S1. These analyses indicate that a smaller q allows a stronger evolutionary response of x and larger reduction in total cancerous mortality M_C.

However, we also found that, even this value $M_C = 0.0644$ is greater than $M_C = 0.0238$, the total cancerous mortality in the original environment with high mortality ($u = 0.2$). These results suggest that the cancerous mortality would be reduced by the evolutionary adjustment of the genomic error rate, and this response is stronger for a small q. However, the reduced fraction of cancerous mortality was still larger than the value in the original population with a high noncancerous mortality.

B.2 Effect of step number n

The step number n differs between cancer types. In general, solid tumors tend to have a large n, whereas leukemia tends to have smaller n. In the standard case, we adopted $n = 5$, which corresponds to a solid tumor. In this section, we provide the results for the evaluation of the effect of step number on the outcome of the total cancerous mortality.

Consider the case in which the step number is the minimum value, $n = 1$, implying that a single event leads to the development of malignant cancer in a patient. To facilitate the comparison with the standard case, we choose $k = 0.0488$ so that the total cancerous mortality in the original environment ($u = 0.2$) is the same: $M_C = 0.0238$. We also maintained the same value of the original genomic error rate, $x = 0.1$. Thus, we found M_C increases when the mortality is improved to $u = 0.0667$ with x remaining unchanged. In Fig. S2a, the curve labeled as "before adaptation" indicates the value of M_C when u is reduced to the value in the horizontal axis, when the step number is $n = 1$. The magnitude of the enhancement of M_C is smaller than in the case of $n = 5$ (compare it with Fig. 4c).

Next, we consider the adaptive evolution of the genomic error rate x. To facilitate the situation to become comparable to the standard case, we adjusted f_0 so that the ESS is $x = 0.1$, the same value as in the standard case. Thus, $f_0 = 0.0168$. Subsequently, we

calculated the ESS value of the genomic error rate x for different values of u. The results are indicated as the curve labeled as "after adaptation" in Fig. S2a. We can see that this curve is considerably lower than the corresponding curve in Fig. 4c for the standard case ($n = 5$).

We also performed a similar analysis for $n = 3$. The results were between the results for $n = 1$ and $n = 5$ (see Fig. S2b). To compare the results, we focused on the values when $u = 0.0667$, and listed the results in Table S2.

We can conclude that, when the step number n is smaller, the total cancerous mortality becomes larger in the improved environment than the original value, but the magnitude of the enhancement was not as large as in the cases with larger n. This result is plausible because the enhancement of the total cancerous mortality would be caused by the people who reach older ages.

We also found that evolutionary adaptation should further reduce the total cancerous mortality, even for the cases with small step numbers.

This also suggests us that enhanced cancerous mortality observed for companion animals (probably for humans as well) should be more important for solid tumors (with large n) than for leukemia (with small n).

B.3 Transition rate becomes faster as the step proceeds

In the standard case, the rate of transition between states is the same for all transitions. However, there are many reasons for the first few transitions to require a considerably longer waiting time than the later steps. To determine the effect of the accelerating rate of transition, we studied a model in which the transition rates follow a geometric sequence: $k_j = k_0 r^j$ $(j = 0, 1, 2, \ldots, n-1)$ where $r > 1$. The standard case corresponds to $r = 1$.

When $r = 5$, we adjusted the value of k_0 to make $M_C = 0.0238$ when $x = 0.1$ and $u = 0.2$. We found $k_0 = 0.2205$. Then we made u smaller than 0.2 and found that M_C increased. In Fig. S3, the results are illustrated by a curve labeled as "$r = 5$" and "before adaptation". The curve labeled as "$r = 1$" and "before adaption" is the same as the one in Fig. 4c. We can see that the curve with $r = 5$ (an accelerating transition speed) is below that with equal transition rates ($r = 1$).

We then considered the evolutionary change in x. We searched for f_0 that makes $x = 0.1$ as the ESS in the original environment ($u = 0.2$). It turned out to be $f_0 = 0.032$. We then calculated the ESS x^* and the corresponding total cancerous mortality M_C. The results for different u are indicated by a curve labeled as "$r = 5$" and "after adaptation". We found that it is below the curve labeled as "$r = 1$" and "after adaptation", which is the same as the one in Fig. 4c. Both before adaptation and after adaptation, M_C with accelerating transition rates ($r = 5$) was lower than that with equal transition rates ($r = 1$). The direction of these shifts is the same as the results for a smaller step number n (illustrated in Fig. S2). We can conclude that the accelerating rate of transition between states has an effect similar to the reduction in the step number.

B.4 Fertility depends on the age

In the standard case discussed in the main text, fertility was assumed to be independent of age a. A more realistic assumption would be that fertility is low both in very young age and old age, and is high in the intermediate values of a. Herein, we assume that

$$\begin{aligned} &m_0(a) = 0 && \text{if } 0 \le a \le 1, \\ &m_0(a) = m_{max}(a-1) && \text{if } 1 < a \le 2, \\ &m_0(a) = m_{max} && \text{if } 2 < a \le 6, \\ &m_0(a) = m_{max}\,(10-a)/4 && \text{if } 6 < a \le 10, \\ &m_0(a) = 0 && \text{if } a > 10 \quad . \end{aligned} \tag{B.1}$$

This imitates the situation of companion dogs, which have the highest fertility from 2 years to 6 years of age. In equation (9), fertility is simply m_0 minus f_0/x^q, the cost for reducing x. If we simply replaced m_0 in equation (9) by function (B.1), the fertility was negative for some ages. To avoid this problem, we may replace $m_0 - f_0/x^q$ in equation (9) by the following quantity

$$m_0(a)\left(1 - \frac{f_0/m_{max}}{x^q}\right) \quad . \tag{B.2}$$

Note that this fertility expression is the same as the one in equation (9) if $m_0(a) = m_{max}$, and it is either positive or zero if we use equation (B.1).

By using this $m_0(a)$ and q, we searched for the value of f_0 that makes $x = 0.1$ to be the ESS under $u = 0.2$. We found that f_0 needs to be very small ($f_0 = 0.0080$). Nest, we calculated the ESS x for a given value of u ($u < 0.2$). The results are shown in Fig. S4a. The evolutionary response of genomic error rate was notable for the improved environment (i.e. smaller u), but the magnitude of the response was very small. This is because the natural selection acting to remove the cancerous mortality is weak, because most of cancerous mortality occurs in the advanced ages where the fertility is zero.

We also calculated the total cancerous mortality M_C. The M_C after adaptation is smaller than that before adaptation (Fig S4b). However, the magnitude of the reduction is very small.

The genomic error rate after adaption under $u = 0.0667$ was $x^* = 0.0932$, which is not much different from $x^* = 0.1$, the ESS value under $u = 0.2$. The total cancerous mortality was $M_C = 0.1875$, which is again close to the one before adaptation: $M_C = 0.2068$. We can conclude that the evolutionary response of the population to the improved environment (i.e. smaller u) is weak and the cancerous mortality remains high, probably because the natural selection acting to remove cancer is weak.

Table S1 Effect of q on the total cancerous mortality M_C. The other parameters were $m_0 = 1.0$, and $n = 5$.

(a)

$q = 0.5$	Original environment	Improved environment	After adaptation
u	0.2	0.0667	0.0667
x	0.1	0.1	0.0424
M_C	0.0238	0.2068	0.0433

$k = 1.8,\ f_0 = 0.36.$

(b)

$q = 1.1$	Original environment	Improved environment	After adaptation
u	0.2	0.0667	0.0667
x	0.1	0.1	0.0507
M_C	0.0238	0.2068	0.0644

$k = 1.8,\ f_0 = 0.044.$

(c)

$q = 2.0$	Original environment	Improved environment	After adaptation
u	0.2	0.0667	0.0667
x	0.1	0.1	0.0599
M_C	0.0238	0.2068	0.0900

$k = 1.8,\ f_0 = 0.0031.$

Table S2 Effect of step number n on the total cancerous mortality M_C. The other parameters were $m_0 = 1.0$, and $q = 1.1$.

(a)

$n = 1$	Original environment	Improved environment	After adaptation
u	0.2	0.0667	0.0667
x	0.1	0.1	0.0602
M_C	0.0238	0.0681	0.0422

$k = 0.0488,\ f_0 = 0.0168.$

(b)

$n = 3$	Original environment	Improved environment	After adaptation
u	0.2	0.0667	0.0667
x	0.1	0.1	0.0520
M_C	0.0238	0.164	0.0577

$k = 0.808,\ f_0 = 0.0360.$

(c)

$n = 5$	Original environment	Improved environment	After adaptation
u	0.2	0.0667	0.0667
x	0.1	0.1	0.0507
M_C	0.0238	0.2068	0.0644

$k = 1.8,\ f_0 = 0.044.$

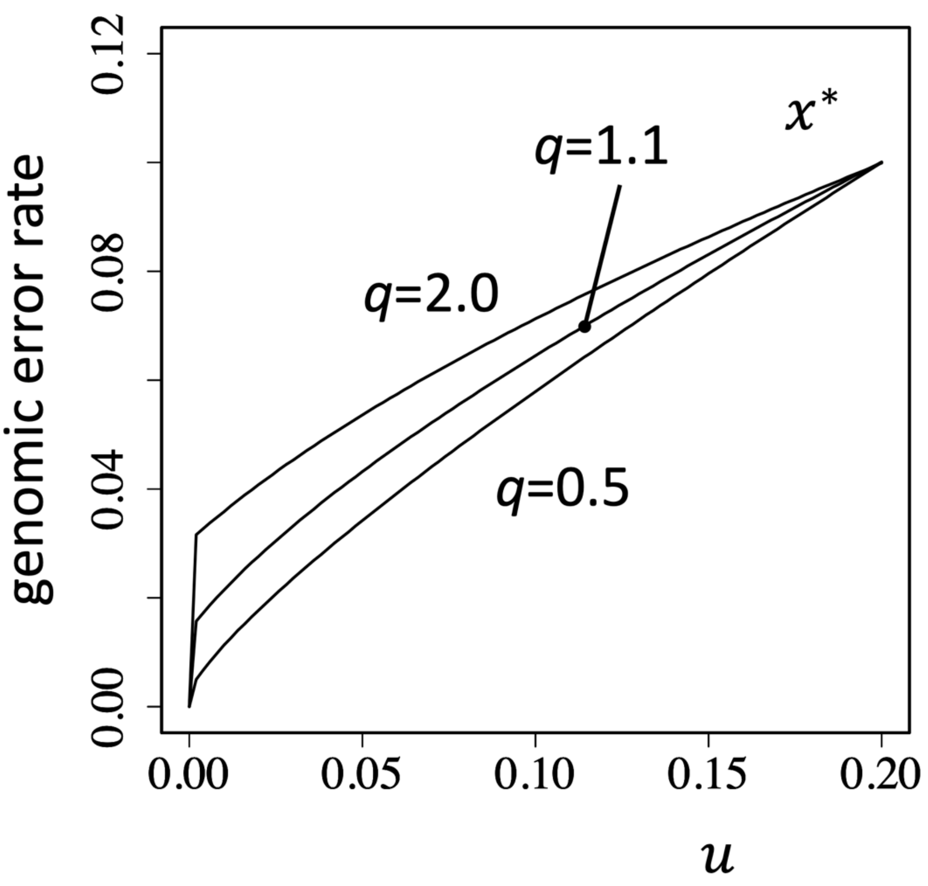

Fig. S1 The ESS genomic error rate x^* for different q. The horizontal axis represents noncancerous mortality u. Three curves are the ESS x^* for different values of q. As the environmental condition is improved, the noncancerous mortality u decreases, and the ESS x^* becomes smaller. The magnitude of this evolutionary response is stronger for a small q than for a large q. The parameters and the method to obtain these curves are explained in Appendix B.

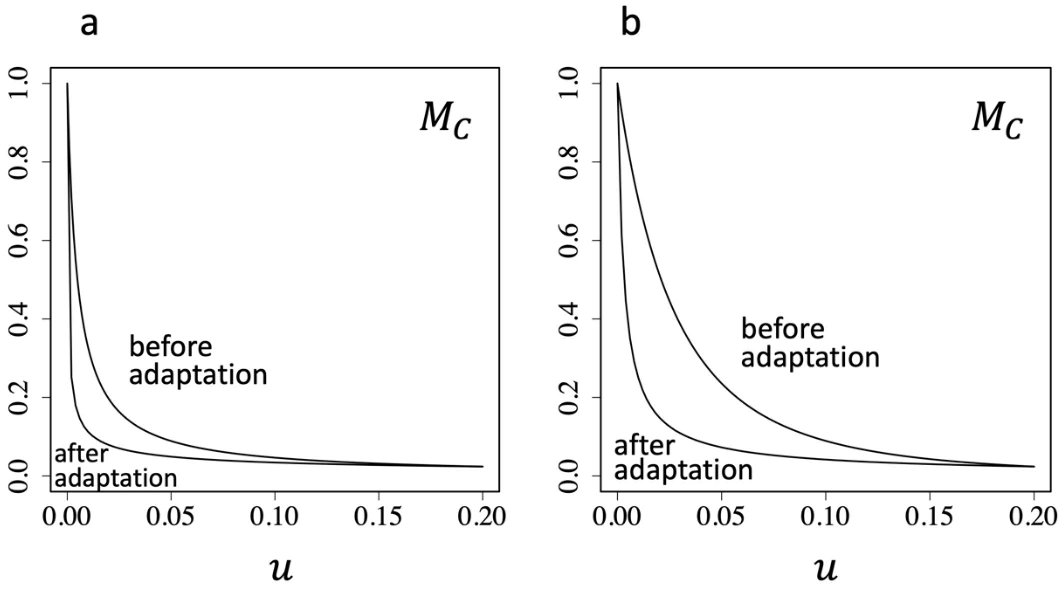

Fig. S2 The total cancerous mortality M_C for different smaller step numbers n. The horizontal axis represents noncancerous mortality u. We show the response of M_C to the reduced u for step numbers smaller than in the standard case. (a) Step number is $n = 1$. (b) Step number is $n = 3$. Explanations are provided in Appendix B.

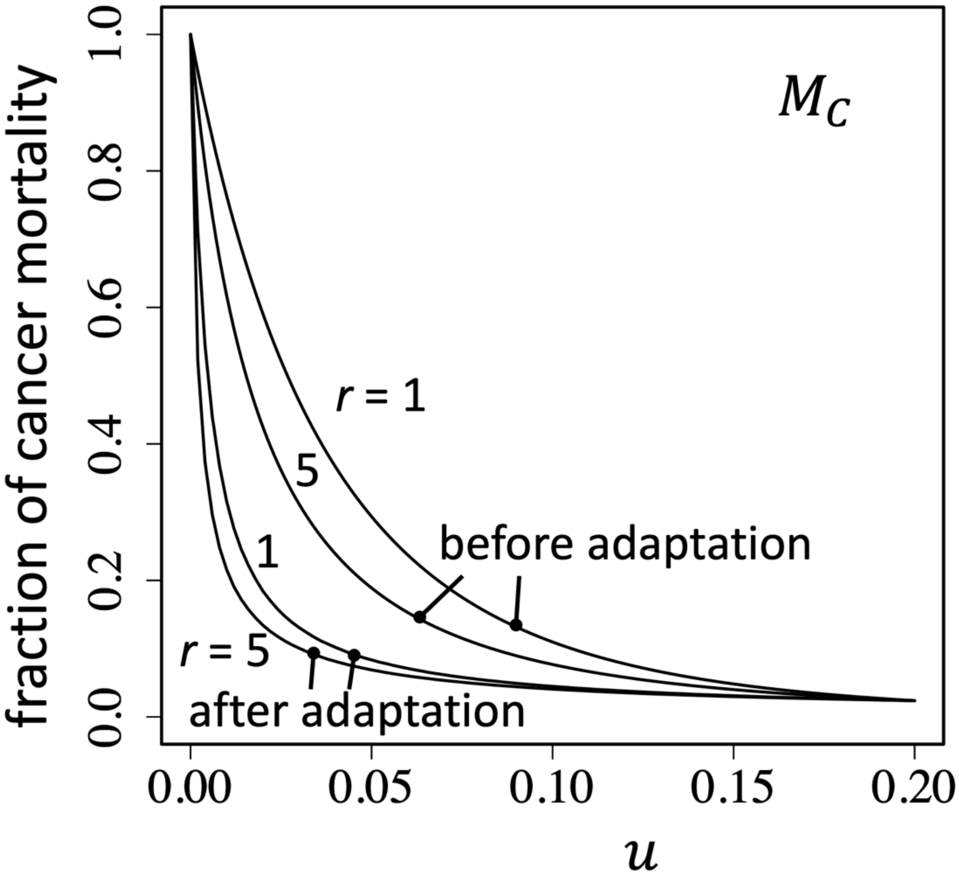

Fig. S3 The total cancerous mortality for accelerating transition rates. The two curves labeled as "$r = 1$" are the same as those in Fig. 4c. The other two curves labeled as "$r = 5$" are the results when the transition rate becomes faster as the step proceeds. The procedure of calculation is explained in Appendix B. Parameters are as follows: $k_0 = 0.2205$ and $f_0 = 0.032$. Other parameters are the same as in the standard case: $m_0 = 10$, $q = 1.1$, and $n = 5$.

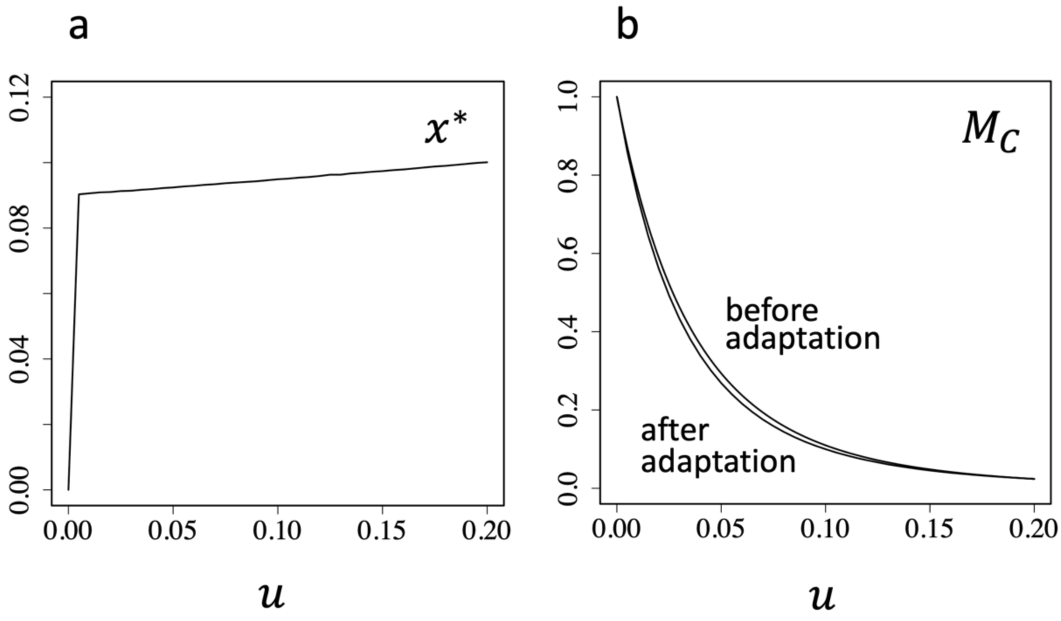

Fig. S4 The evolutionary responses when the fertility depends on age. (a) The ESS genomic error rate x^* for different noncancerous mortality u. When u became smaller, the ESS genomic error rate x^* decreased, but the magnitude of reduction was considerably smaller than that in the standard case (see Fig. 4b). (b) Total cancerous mortality M_C after adaptation of genomic error rate. M_C also decreased by the evolutionary adaptation, but the magnitude of reduction was smaller than that in the standard case of age-independent fertility. See Appendix B for explanations.
